# Plant specimen contextual data consensus

**DOI:** 10.1093/gigascience/giw002

**Published:** 2016-12-29

**Authors:** Petra ten Hoopen, Ramona L. Walls, Ethalinda KS Cannon, Guy Cochrane, James Cole, Anjanette Johnston, Ilene Karsch-Mizrachi, Pelin Yilmaz

**Affiliations:** 1 European Molecular Biology Laboratory, European Bioinformatics Institute, Wellcome Genome Campus, Hinxton, Cambridge CB10 1SD, United Kingdom; 2 CyVerse, University of Arizona, Tucson, Arizona, USA; 3 Department of Computer Science, Iowa State University, Ames, Iowa, USA; 4 United States Department of Agriculture–Agricultural Research Service (USDA–ARS), Corn Insects and Crop Genetics, Ames, Iowa, USA; 5 Department of Plant, Soil and Microbial Sciences, Michigan State University, East Lansing, Michigan 48824, USA; 6 National Center for Biotechnology Information, National Library of Medicine, National Institutes of Health, Building 38A, 8600 Rockville Pike, Bethesda, Maryland 20894, USA; 7 Microbial Genomics and Bioinformatics Research Group, Max Planck Institute for Marine Microbiology, Celsius str. 1, Bremen 28359, Germany

**Keywords:** Plant, Specimen, Contextual data, Checklist

## Abstract

The Compliance and Interoperability Working Group of the Genomic Standards Consortium facilitates the establishment of a community of experts and the development of recommendations to describe genomic data and associated information. Here we present our ongoing conation to harmonise the reporting of contextual plant specimen data associated with genomics and functional genomics. This commentary summarises the current state of our plant sample contextual data harmonisation efforts to engage a broad plant science community.

## Background

Publishing well-structured data in an established data resource supports the discoverability and safe preservation of legacy data. If related contextual data is structured in a similar manner, further scientific advances may be made by meaningful comparisons of data sets.

Data and contextual data standards bring structure to data, providing recommendations on data formats and specifying attributes that categorise the data. However, standardisation efforts should be harmonised to prevent duplicating work or establishing contradictory practices.

With extensive global interest in the molecular analysis of plant species for food, forestry, biomass and other applications, we have entered an era of extensive publication of plant molecular data sets in need of structure. For example, 0.37 terabases of plant assembled sequence data from 1017 studies were presented in International Nucleotide Sequence Database Collaboration (INSDC) databases to August 2016.

Here we describe an ongoing endeavour to harmonise recommendations to support the plant science community in reporting plant specimen contextual data associated with genomic and functional genomic experiments to data archives.

## Plant specimen contextual data consensus

Plant specimen contextual data provides information about the plant material being analysed in a molecular assay. This information layer is distinct from the investigation layer, which specifies the purpose of the investigation and its authors; and from the experiment layer, which describes the molecular experiment design. Plant specimen contextual information is also independent of the plant molecular analysis, meaning that a common set of plant specimen descriptors can be used to report the contextual information about a plant sample associated with a molecular data set.

**Fig. 1:**
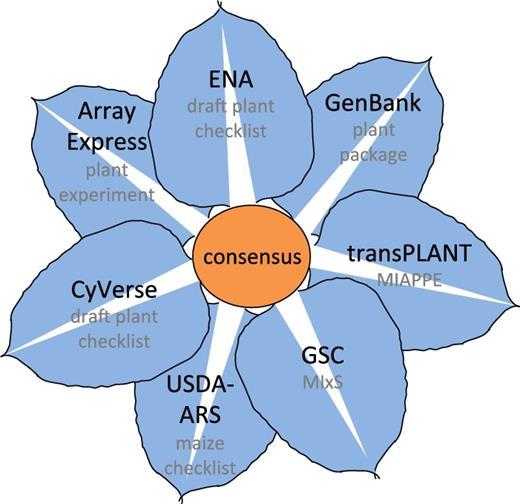
Data resources/initiatives and guidelines contributing to development of the Plant Specimen Contextual Data Consensus. Data resources or initiatives are shown clockwise and in black; guidelines are in grey. Array Express & Expression Atlas and European Nucleotide Archive (ENA) & BioSamples at the European Molecular Biology Laboratory, European Bioinformatics Institute (EMBL-EBI, UK); GenBank & BioSample at the National Center for Biotechnology Information (NCBI, USA); transnational transPLANT and Genomic Standards Consortium (GSC); Agricultural Research Service of the US Department of Agriculture (USDA-ARS, USA); and CyVerse (USA).

Several projects are developing recommendations for the reporting of plant molecular or phenotyping data (Fig. 1). We aim to unify these developments in a common contextual data set – the Plant Specimen Contextual Data Consensus – that will contribute to the consistent reporting of plant specimen information to data repositories and improve the integration of specimen-associated molecular data among repositories.

Several resources were involved in this exercise: 1) the European Nucleotide Archive (ENA) and BioSamples at the European Molecular Biology Laboratory-European Bioinformatics Institute (EMBL-EBI), UK [1], which archives genomic and transcriptomic data and associated contextual data; 2) CyVerse (formerly the iPlant Collaborative), USA [2], a computation infrastructure for life sciences; 3) GenBank [3] and BioSample databases at the National Center for Biotechnology Information (NCBI), USA [4], which archives nucleotide sequence data and associated sample contextual information; and 4) the United States Department of Agriculture (USDA) Agricultural Research Service (ARS), USA, a scientific research agency for agriculture. We have also drawn from the expertise of Array Express [5] and Expression Atlas [6] at the EMBL-EBI, UK to collect plant transcriptomic data and received valuable input from authors of the Minimum Information about a Plant Phenotyping Experiment (MIAPPE) standard developed by transPLANT [7], a transnational project to construct an e-infrastructure for plant genomics. Furthermore, we reused several concepts specified in the core and plant host-associated environmental package of the Minimum Information about any (x) Sequence (MIxS) standard [8] developed by the Genomic Standards Consortium (GSC) [9].

To develop the Plant Specimen Contextual Data Consensus, independent plant checklists drafted at ENA, USDA-ARS and CyVerse were mapped to the plant package developed at GenBank and the plant host-associated MIxS environmental package. Duplications were removed, and descriptor names, definitions and the use of ontologies were harmonised. This merged draft was then shared with plant communities associated with CyVerse, Array Express and developers of the MIAPPE standard for comments. A new merged draft incorporating comments from this consultation was created and re-reviewed by all co-authors, covering content and descriptor groupings and recommendations for the level of requirement of each descriptor. Final amendments resulted in the mature first version of the Consensus, which co-authors formally published to enable it to be used and further refined by a wider plant science community.

Deciding the scope and requirement level of Consensus descriptors was a challenge in this process: having too few descriptors would not fulfil plant experts’ expectations, but having too many requirements could prevent its adoption. For instance, inflation of plant phenotypic characteristics would lead to granularity exceeding generic usage of the Consensus.

Another challenge concerned compliance to existing standards: the Plant Specimen Contextual Data Consensus Version 1.0 is not fully compliant to the existing MIxS standard since some minimum information descriptors in MIxS are well suited to microorganisms but not so relevant to evolutionarily higher organisms. Discussion on a possible solution to establish existing standard profiles is beyond the scope of this publication.

The Plant Specimen Contextual Data Consensus Version 1.0 is available in Supplementary Table S1. However, the Consensus is likely to evolve and we therefore encourage readers to view the latest version at the GSC website [10]. Each contextual data attribute of the Consensus is described with a name, category, suggested requirement level (M: mandatory; C: recommended; X: optional), definition, format and mapping to an available ontology class.

Descriptors are divided into four categories:

1. Organism descriptors specify taxonomic information;
2. Sample descriptors characterise the material taken from the plant organism and used for an experiment;
3. Treatment descriptors describe the plant's natural and imposed environmental conditions before the sample was taken;
4. Growth medium descriptors provide details of the plant rooting conditions.

The Consensus recommends several established relevant ontologies and controlled vocabularies: Plant Ontology (PO), Phenotypic Quality Ontology (PATO), Crop Ontology (CO), Plant Trait Ontology (TO), Plant Environment Ontology (EO), Environment Ontology (ENVO), Experimental Factor Ontology (EFO), Chemical Entities of Biological Interest (CHEBI) the NCBI Taxonomy index and INSDC country controlled vocabulary.

Ten Consensus descriptors were identified as essential contextual data for a plant sample of any molecular experiment; these are highlighted in bold and suggested as mandatory. Moreover, a subset of recommended descriptors may be considered depending on the experiment or implementation. Optional descriptors offer further reporting granularity.

Although practical implementation of the Consensus might vary depending on the resource adopting it, the Consensus offers the potential for plant specimen contextual data to be harmonised across molecular assays. One implementation is available in the ENA’s data submission system for the deposition of plant genomic and transcriptomic data to INSDC. This can add to the collection of well-described plant samples, such as the *Brassica oleracea* sample SAMN03858113 or the *Hordeum vulgare* sample SAMN04549447.

The Consensus presented here is largely in line with recommendations for the description of a plant bioresource and its environment and treatment formulated for plant phenotyping data [7]. We also envisage that it may be used to describe samples associated with metabolic data.

## Conclusion

With the current substantive need to integrate data beyond scientific domains and political borders, it is fundamental for both short-term and long-term initiatives to unite forces when working towards similar goals. Presented here is an example of an ongoing transatlantic community collaboration (Fig. 1) with a common goal to provide plant scientists with recommendations on how to describe plant specimens analysed in molecular experiments. This can contribute to the consistent description of plant specimens and improve integration of specimen-associated molecular data.

## Additional file

Supplementary data are available at *GIGSCI* online.

**Additional file 1: Table S1.** The Plant Specimen Contextual Data Consensus. Each concept of the Consensus is described with a name, category, suggested requirement level (M: mandatory; C: recommended; X: optional), definition, format and mapping to an available ontology class. (DOCX 119 kb)

## Abbreviations

CHEBI: Chemical Entities of Biological Interest
CO: Crop Ontology
EFO: Experimental Factor Ontology
EMBL-EBI: European Molecular Biology Laboratory, European Bioinformatics Institute
ENA: European Nucleotide Archive
ENVO: Environment Ontology
EO: Plant Environment Ontology
GSC: Genomic Standards Consortium
INSDC: International Nucleotide Sequence Database Collaboration
NCBI: National Center for Biotechnology Information
MIAPPE: Minimum Information about a Plant Phenotyping Experiment
MIxS: Minimum Information about any (x) Sequence
PATO: Phenotypic Quality Ontology
PO: Plant Ontology
TO: Plant Trait Ontology
USDA-ARS: Agricultural Research Service of the US Department of Agriculture

## Availability of data and material

All data generated during this study are included in this published article and its supplementary information files.

## Competing interests

The authors declare that they have no competing interests.

## Funding

This work was supported by: the Biotechnology and Biological Sciences Research Council award BB/M018458/1 to EMBL-EBI for GC and PTH; National Science Foundation award numbers DBI-0735191 and DBI-1265383 to CyVerse for RLW; and in part by the Intramural Research Program of the National Institutes of Health, National Library of Medicine for IKM and AJ.

## Authors’ contributions

PTH coordinated harmonisation of the plant specimen contextual data, drafted the ENA plant checklist and mapped the draft to the Array Express, transPLANT and MIxS checklists. RLW and EKSC led on mapping to the plant contextual data checklists developed at CyVerse and USDA-ARS, respectively. AJ led on mapping to the NCBI Plant Package. IKM, GC and JC provided overall guidance. PY advised on MIxS standard concepts and published the Plant Specimen Contextual Data Consensus on the GSC website. PTH wrote the manuscript with an editorial contribution and a revision by all co-authors. All authors read and approved the final manuscript.
