# Supplementary material for: Plant specimen contextual data consensus
Source: Gigascience. 2016 Dec 29;5(1):1–4. doi: 10.1093/gigascience/giw002 (PMC5572840; doi:10.1093/gigascience/giw002)
Supplement: Supplementary file 1 [file giw002_Supp.docx]

| **Consensus term** | **Category** | **Requirement level** | **Description** | **Ontology class** |
| --- | --- | --- | --- | --- |
| taxid | organism | M | NCBI taxon ID of the subject, e.g. 9606. | NCBI taxon identifier (APOLLO_SV_00000203) |
| organism common name | organism | C | Common name of the subject organism, e.g. maize. |  |
| subspecific genetic lineage  rank | organism | C | Further information about the genetic distinctness of this lineage by recording additional information, i.e. variety, cultivar, ecotype, inbred line. It can also contain alternative taxonomic information. | cultivar (EFO_0005136), ecotype (EFO_0000434) |
| subspecific genetic lineage  name | organism | C | Name of the infraspecific rank, e.g. ecotype Col-0. |  |
| ploidy | organism | C | The ploidy level of the genome (e.g. allopolyploid, haploid, diploid, triploid, tetraploid). For terms, please select terms listed under class ploidy (PATO:001374) of Phenotypic Quality Ontology (PATO), and for a browser of PATO (v 2013-10-28) please refer to <http://purl.bioontology.org/ontology/PATO>. | ploidy (EFO_0000659), ploidy (PATO_0001374) |
| extrachromosomal elements | organism | X | Any extra or missing chromosomes or extra-chromosomal elements. |  |
| number of replicons | organism | C | The number of replicons in a nuclear eukaryotic genome, always applied to the haploid chromosome count of a eukaryote. |  |
| estimated size | organism | C | The estimated haploid genome size prior to sequencing, of particular importance in the sequencing of eukaryotic genomes that might remain in draft form for a long or unspecified period. |  |
| genotype | organism | X | Name or code for genotype of the organism, e.g. C1 Bz1/c1 bz1, which encodes important alleles present in the stock. | genotype (EFO_0000513) |
| biological status | organism | X | The level of genome modification; controlled vocabulary: wild, natural, semi-natural, inbred line, breeder's line, hybrid, clonal selection, mutant. |  |
| genetic modification | organism | X | Genetic modifications of the genome of an organism, which may occur naturally by spontaneous mutation, or be introduced by some experimental means, e.g. specification of a transgene or the gene knocked-out or details of transient transfection. | genetic modification (EFO_0000510) |
| organism phenotype | organism | X | Most relevant phenotypic traits of the subject. For Phenotypic Quality Ontology (PATO) (v 2013-10-28) terms, please see <http://purl.bioontology.org/ontology/PATO>, e.g. bifurcated (PATO_0001784). Terms from Trait Ontology (TO), Plant Ontology (PO) or Crop Ontology (CO) are also accepted; include name/method/scale for each trait; can include multiple traits. | phenotype (EFO_0000651) |
| ancestral data | organism | X | Information about either pedigree or other ancestral information description (e.g. parental variety in case of mutant or selection), e.g. A/3*B (meaning [(A x B) x B] x B). |  |
| source material identifiers | organism | C | A unique identifier assigned to a material sample (as defined by <http://rs.tdwg.org/dwc/terms/materialSampleID>, and as opposed to a particular digital record of a material sample) used for extracting nucleic acids, and subsequent sequencing, the identifier can refer either to the original material collected or to any derived sub-samples. | specimen identifier assigned by specimen repository (OBI_0001900) |
| source material description | organism | X | Further information to clarify the nature of the specimen or population used that is not collected elsewhere, e.g. if the source was derived from accessioned stock, describe how it links to the original material. |  |
| biotic relationship | organism | X | Free text description of relationship(s) between the subject organism and other organism(s) it is associated with, e.g., parasite on species X; mutualist with species Y, the target organism is the subject of the relationship, and the other organism(s) is the object. |  |
| growth habit | organism | X | Characteristic shape, appearance or growth form of a plant species; controlled vocabulary: erect, semi-erect, spreading, prostrate. | shoot habit (TO_0002756) |
| propagation | organism | C | Sexual or asexual propagation of the specimen. |  |
| plant sex | organism | X | Sex of the reproductive parts on the whole plant, e.g. pistillate, staminate, monoecieous, hermaphrodite. | pistillate flower (PO_0025599), staminate flower (PO_0025600) |
| sample name | sample | M | Unique name for the sample from an organism within the data set, e.g. Zea1564. |  |
| sample title | sample | M | Brief informative sample title, e.g. ‘shoot apical meristem of *Zea mays*, variety Hobbit’. |  |
| biosample accession | sample | C | Unique permanent identifier issued by the INSDC Databases. |  |
| sample description | sample | X | Brief description of the sample. | sample description (sep_00196) |
| sample capture status | sample | X | Reason for the sample; controlled vocabulary: active surveillance in response to an outbreak, active surveillance not initiated by an outbreak, farm sample, market sample, other. |  |
| growth facility | sample | C | Type of facility where the sampled plant was grown; controlled vocabulary: growth chamber, open top chamber, glasshouse, experimental garden, field. Alternatively use Crop Ontology (CO) terms. |  |
| isolation and growth condition | sample | M | Publication reference in the form of PubMed ID (pmid), digital object identifier (doi) or url for isolation and growth condition specifications of the sample. | growth condition (EFO_0000523) |
| sample collection device or method | sample | X | Method or device employed for collecting the sample. | sample collection protocol (EFO_0005518) |
| sample material processing | sample | X | Any processing applied to the sample during or after retrieving the sample from environment, this field accepts OBI, for a browser of OBI (v 2013-10-25) terms please see <http://purl.bioontology.org/ontology/OBI>. | biological sample processing (ERO_0000705) |
| amount or size of sample collected | sample | X | Amount or size of sample (volume, mass or area) collected. | sample volume (sep_00197) |
| plant structure | sample | M | Name of plant structure the sample was obtained from; for Plant Ontology (PO) (v 20) terms, see <http://purl.bioontology.org/ontology/PO>, e.g. petiole epidermis (PO_0000051). If an individual flower is sampled, the sex of it can be recorded here. | plant structure (PO_0009011) |
| developmental stage | sample | M | Developmental stage at the time of sample collection; for Plant Ontology (PO) (v 20) terms, see <http://purl.bioontology.org/ontology/PO>, e.g. hypocotyl emergence stage (PO_0007043). | plant structure development stage (PO_0009012) |
| sampled age | sample | X | Age of subject at the time of sample collection; relevant scale depends on species and study; e.g. 2 weeks old. | age (EFO_0000246) |
| sample phenotype | sample | X | Phenotype of the plant from which the sample was obtained, such as colour of corolla, fruit diameter, circular leaf shape. Plant Trait Ontology (TO), Phenotypic Quality Ontology (PATO), or other ontology is recommended; e.g. stem epidermis colour (TO:1000018): light green. | portion of plant tissue morphology trait (TO_0000843) |
| sample health state | sample | C | Health status of the subject at the time of sample collection, controlled vocabulary: diseased or healthy. |  |
| sample disease status | sample | C | List of diseases with which the subject has been diagnosed at the time of sample collection; can include multiple diagnoses; the value of the field depends on subject; e.g. Charcoal rot (*Macrophomina phaseolina*), Late wilt (*Cephalosporium maydis*). | disease (EFO_0000408) |
| sample disease stage | sample | X | Stage of the disease at the time of sample collection, e.g. inoculation, penetration, infection, growth and reproduction, dissemination of pathogen. | disease staging (EFO_0000410) |
| sample wet mass | sample | X | Measurement of wet mass at the time of sample collection; e.g. 0.23 g. | plant fresh weight (TO_0000442) |
| sample dry mass | sample | X | Measurement of dry mass at the time of sample collection; e.g. 0.05 g. | plant dry weight (TO_0000352) |
| sample height | sample | X | Height of subject at the time of sampling, if different from the length; e.g. 0.75 m. | plant height (TO_0000207) |
| sample length | sample | X | Length of subject at the time of sampling, if different from the height; e.g. 2 m. | length (PATO_0000122) |
| sample storage duration | sample | X | Period of time for which the sample was stored; e.g. 3 months. |  |
| sample storage location | sample | X | Location at which sample was stored, usually name of a specific freezer/room. |  |
| sample storage temperature | sample | X | Temperature at which sample was stored, e.g. −80 degrees Celsius. | storage temperature setting (OMIABIS_0001013) |
| sample volume or weight for DNA extraction | sample | X | Volume (mL) or weight (g) of the sample processed for DNA extraction. |  |
| geographic location (latitude and longitude) | sample | M | Geographical origin of the sample as defined by latitude and longitude; the values should be reported in decimal degrees and in WGS84 system. | collection latitude (EFO_0005020), collection longitude (EFO_0005021) |
| geographic location (country, sea, region) | sample | M | Geographical origin of the sample as defined by the country or sea name, followed by specific region name. Country or sea names should be chosen from the INSDC country list (<http://insdc.org/country.html>), or the GAZ ontology (v 1.512): <http://purl.bioontology.org/ontology/GAZ>. | national geopolitical entity (ENVO_00000009) |
| geographic location (depth) | sample | X | Vertical distance below local surface; can be reported as an interval for subsurface samples. | verbatimDepth |
| geographic location (altitude) | sample | X | Vertical distance between Earth’s surface above sea level and the sampled position in the air. | altitude (SIO_000438) |
| geographic location (elevation) | sample | X | Vertical distance from mean sea level. | verbatimElevation |
| collection date | sample | M | Time of sampling, either as an instance (single point in time) or interval. In case no exact time is available, the date/time can be right-truncated; e.g. all of these are valid times: 2008-01-23T19:23:10+00:00; 2008-01-23T19:23:10; 2008-01-23; 2008-01; 2008. Except 2008-01; 2008 all are ISO8601 compliant. | specimen collection time measurement (OBI_0001619) |
| sampling time point | sample | X | Time point(s) at which a sample or observation is made or taken from a biomaterial as measured from some reference in a day, month, or other period; e.g. 9:00, 14:00, 19:00, 24:00; or first and 15th day of the month. This field is used to record the specific time point(s) at which an organism is sampled within the range listed in collection date. | time point (EFO_0000724) |
| environment (biome) | sample | X | Biomes are defined based on factors such as plant structures, leaf types, plant spacing, and other factors like climate. Biome should be treated as the descriptor of the broad ecological context of a sample, e.g. desert, taiga, deciduous woodland, or coral reef. EnvO (v 2013-06-14) terms can be found via the link: [www.environmentontology.org/Browse-EnvO](http://www.environmentontology.org/Browse-EnvO). | biome (ENVO_00000428) |
| environment (feature) | sample | X | Environmental feature level includes geographic environmental features. Compared to ‘biome’, ‘feature’ is a descriptor of the more local environment, e.g. harbour, cliff, or lake. EnvO (v 2013-06-14) terms can be found via the link: [www.environmentontology.org/Browse-EnvO](http://www.environmentontology.org/Browse-EnvO). | environmental feature (ENVO_00002297) |
| collected by | sample | X | Name of person(s) or institute who collected the specimen; e.g. John Smiths, Jane White. |  |
| identified by | sample | X | Name of person(s) who identified the specimen taxonomically, e.g. David Green. | IdentifiedBy |
| treatment | treatment | C | Ontology term(s) describing the plant treatment or relevant environmental conditions; recommend use of Plant Environment Ontology (EO) or other ontology, such as XEML Environment Ontology (XEO) or Crop Ontology (CO). More specific fields in the treatment section can be used in addition to or in place of this field. | plant treatment (EO_0001001) |
| air temperature regimen | treatment | C | Information about treatment(s) involving exposure to varying temperatures; should include the temperature, treatment duration, interval and total experimental duration; can include different temperature regimens. | air temperature regimen (EO_0007161) |
| antibiotic regimen | treatment | X | Information about treatment(s) involving antibiotic administration; should include the name of antibiotic, amount administered, treatment duration, interval and total experimental duration; can include multiple antibiotic regimens. | antibiotic treatment (EO_0007041) |
| chemical administration | treatment | X | List of chemical compounds administered to the host or site where sampling occurred, e.g. N fertilizer. Can include multiple compounds. For Chemical Entities of Biological Interest ontology (CHEBI) (v 111) see <http://purl.bioontology.org/ontology/CHEBI>. | chemical treatment (EO_0007189) |
| chemical mutagen | treatment | X | Treatment involving use of mutagens; should include the name of mutagen, amount administered, treatment duration, interval and total experimental duration; can include multiple mutagen regimens. | chemical mutagen treatment (EO_0007149) |
| climate environment | treatment | X | Treatment involving an exposure to a particular climate; can include multiple climates. | ecological environment (EO_0007064) |
| fertilizer regimen | treatment | X | Information about treatment(s) involving the use of fertilizers; should include the name fertilizer, amount administered, treatment duration, interval and total experimental duration; can include multiple fertilizer regimens. | fertilizer regimen (EO_0007085) |
| fungicide regimen | treatment | X | Information about treatment(s) involving use of fungicides; should include the name of fungicide, amount administered, treatment duration, interval and total experimental duration; can include multiple fungicide regimens. | fungicide treatment (EO_0007268) |
| gaseous environment | treatment | X | Use of conditions with differing gaseous environments; should include the name of gaseous compound, amount administered, treatment duration, interval and total experimental duration; can include multiple gaseous environment regimens. | gaseous treatment (EO_0007023) |
| gravity | treatment | X | Information about treatment(s) involving use of gravity factor to study various types of responses in presence, absence or modified levels of gravity; can include multiple treatments. | gravity (EO_0007146) |
| growth hormone regimen | treatment | X | Information about treatment(s) involving use of growth hormones; should include the name of growth hormone, amount administered, treatment duration, interval and total experimental duration; can include multiple growth hormone regimens. | growth hormone treatment (EO_0007165) |
| herbicide regimen | treatment | X | Information about treatment(s) involving use of herbicides; should include the name of herbicide, amount administered, treatment duration, interval and total experimental duration; can include multiple regimens. | herbicide treatment (EO_0007183) |
| humidity regimen | treatment | X | Information about treatment(s) involving exposure to varying degree(s) of humidity; should include amount of humidity administered, treatment duration, interval and total experimental duration; can include multiple regimens. | humidity regimen (EO_0007197) |
| mechanical damage | treatment | X | Information about any mechanical damage exerted on the plant; can include multiple damages and sites. | mechanical damage (EO_0007373) |
| mineral nutrient regimen | treatment | C | Information about treatment(s) involving the use of mineral supplements; should include the name of mineral nutrient, amount administered, treatment duration, interval and total experimental duration; can include multiple mineral nutrient regimens. | nutrient treatment (EO_0007241) |
| non-mineral nutrient regimen | treatment | X | Information about treatment(s) involving the exposure of plant to non-mineral nutrient such as oxygen, hydrogen or carbon; should include the name of non-mineral nutrient, amount administered, treatment duration, interval and total experimental duration; can include multiple non-mineral nutrient regimens. | non-mineral nutrient regimen (EO_0007043) |
| pesticide regimen | treatment | X | Information about treatment(s) involving use of pesticide; should include the name of pesticide, amount administered, treatment duration, interval and total experimental duration; can include multiple pesticide regimens. | pesticide treatment (EO_0007167) |
| pH regimen | treatment | X | Information about treatment(s) involving exposure of plants to varying levels of pH of the growth media; can include multiple regimen. | pH regimen (EO_0007171) |
| radiation regimen | treatment | X | Information about treatment(s) involving exposure of plant or a plant part to a particular radiation regimen; should include the radiation type, amount or intensity administered, treatment duration, interval and total experimental duration; can include multiple radiation regimens. | radiation treatment (EO_0007151) |
| rainfall regimen | treatment | X | Information about treatment(s) involving an exposure to a given amount of rainfall; can include multiple regimens. | rainfall (EO_0007181) |
| salt regimen | treatment | X | Information about treatment(s) involving use of salts as supplement to liquid and soil growth media; should include the name of salt, amount administered, treatment duration, interval and total experimental duration; can include multiple salt regimens. | salt treatment (EO_0007185) |
| seasonal environment | treatment | X | Treatment involving exposure to a particular season (e.g. winter, summer, rabi, rainy etc.). | seasonal environment (EO_0007027) |
| standing water regimen | treatment | X | Treatment(s) involving exposure to standing water during a plant’s life span; types can be flood water or standing water; can include multiple regimens. | standing water (EO_0007282) |
| water temperature regimen | treatment | X | Information about treatment(s) involving exposure to water with varying degrees of temperature; can include multiple regimens. | water temperature regimen (EO_0007160) |
| watering regimen | treatment | C | Information about treatment(s) involving exposure to watering frequencies, for soil top/bottom/drop irrigation; can include multiple regimens. | watering regimen (EO_0007383) |
| perturbation | treatment | X | Type of perturbation, e.g. chemical administration, physical disturbance, etc., coupled with time that perturbation occurred; can include multiple perturbation types. |  |
| light regimen | treatment | C | Information about treatment(s) involving exposure to light, including both light intensity and quality. | light regimen (EO_0007196), light quantity regimen (EO_0007078) |
| biotic regimen | treatment | X | Information about treatment(s) involving use of biotic factors, such as bacteria, viruses or fungi. | biotic plant treatment (EO_0007357) |
| plant growth medium | growth medium | M | Specification of the media for growing the plants or tissue cultured samples, e.g. soil, aeroponic, hydroponic, *in vitro* solid culture medium, *in vitro* liquid culture medium. Recommended value is a specific value from EO:plant growth medium or other controlled vocabulary. | plant growth medium (EO_0007147) |
| rooting conditions | growth medium | C | Relevant rooting conditions such as field plot size, sowing density, container dimensions, number of plants per container. |  |
| culture rooting medium | growth medium | C | Name or reference for the hydroponic or *in vitro* culture rooting medium; can be the name of a commonly used medium or reference to a specific medium, e.g. Murashige and Skoog medium. If the medium has not been formally published, use the rooting medium descriptors. | in vitro growth medium (EO_0007266) |
| rooting medium macronutrients | growth medium | C | Measurement of the culture rooting medium macronutrients (N,P, K, Ca, Mg, S); e.g. KH2PO4 (170 mg/L). | macronutrient (CHEBI:33937) |
| rooting medium micronutrients | growth medium | C | Measurement of the culture rooting medium micronutrients (Fe, Mn, Zn, B, Cu, Mo); e.g. H3BO3 (6.2 mg/L). | micronutrient (CHEBI:27027) |
| rooting medium organic supplements | growth medium | C | Organic supplements of the culture rooting medium, such as vitamins, amino acids, organic acids, antibiotics activated charcoal; e.g. nicotinic acid (0.5 mg/L). | vitamin (CHEBI:33229) |
| rooting medium carbon | growth medium | C | Source of organic carbon in the culture rooting medium; e.g. sucrose. |  |
| rooting medium regulators | growth medium | C | Growth regulators in the culture rooting medium such as cytokinins, auxins, gybberellins, abscisic acid; e.g. 0.5 mg/L NAA. | plant growth regulator (CHEBI:26155) |
| rooting medium solidifier | growth medium | C | Specification of the solidifying agent in the culture rooting medium; e.g. agar. |  |
| rooting medium pH | growth medium | C | pH measurement of the culture rooting medium; e.g. 5.5. |  |
| soil classification FAO | growth medium | C | Soil classification from the FAO World Reference Database for Soil Resources. The list can be found at <http://www.fao.org/nr/land/sols/soil/wrb-soil-maps/reference-groups>, e.g. chernozem. |  |
| soil classification local | growth medium | X | Soil classification based on local soil classification system, e.g. organosol calcaire. |  |
| soil classification method | growth medium | X | Reference or method used in determining the local soil classification. |  |
| soil type | growth medium | X | Soil series name or other lower-level classification (that includes: clay soil, loam soil, sandy loam soil and sandy soil). | soil type (EO_0007155) |
| soil type method | growth medium | X | Reference or method used in determining soil series name or other lower-level classification. |  |
| drainage classification | growth medium | X | Drainage classification from a standard system such as the USDA system; e.g moderately well drained. |  |
| soil texture | growth medium | X | The relative proportion of different grain sizes of mineral particles in a soil, as described using a standard system; express as % sand (50 µm to 2 mm), silt (2 µm to 50 µm), and clay (<2 µm) with textural name (e.g., silty clay loam) optional. | soil texture (EO_0007050) |
| soil texture method | growth medium | X | Reference or method used in determining soil texture. |  |
| soil water content | growth medium | X | Water content measurement; e.g. 13.6%. | soil water content (EO_0007259) |
| soil pH | growth medium | C | pH measurement of the soil; e.g. 6.2. | soil pH environment (EO_0007058) |
